# Alien Domains Shaped the Modular Structure of Plant NLR Proteins

**DOI:** 10.1093/gbe/evz248

**Published:** 2019-11-15

**Authors:** Giuseppe Andolfo, Antimo Di Donato, Pasquale Chiaiese, Antonino De Natale, Antonino Pollio, Jonathan D G Jones, Luigi Frusciante, Maria Raffaella Ercolano

**Affiliations:** 1 Department of Agricultural Sciences, University of Naples “Federico II”, Portici (Naples), Italy; 2 Department of Biology, University of Naples “Federico II”, Italy; 3 The Sainsbury Laboratory, University of East Anglia, Norwich Research Park, NR4 7UH Norwich, United Kingdom

**Keywords:** *R* gene, NLR gene family, Viridiplantae, phylogenetic analysis, horizontal gene transfer, supra domain

## Abstract

Plant innate immunity mostly relies on nucleotide-binding (NB) and leucine-rich repeat (LRR) intracellular receptors to detect pathogen-derived molecules and to induce defense responses. A multitaxa reconstruction of NB-domain associations allowed us to identify the first NB–LRR arrangement in the Chlorophyta division of the Viridiplantae. Our analysis points out that the basic NOD-like receptor (NLR) unit emerged in Chlorophytes by horizontal transfer and its diversification started from Toll/interleukin receptor–NB–LRR members. The operon-based genomic structure of *Chromochloris zofingiensis* NLR copies suggests a functional origin of NLR clusters. Moreover, the transmembrane signatures of NLR proteins in the unicellular alga *C. zofingiensis* support the hypothesis that the NLR-based immunity system of plants derives from a cell-surface surveillance system. Taken together, our findings suggest that NLRs originated in unicellular algae and may have a common origin with cell-surface LRR receptors.

## Introduction

Plant innate immunity relies on two protection levels to prevent or control pathogen infections and requires pathogen detection by either cell surface or intracellular receptors (Dangl and Jones 2001; Jones and Dangl 2006; Jones et al*.* 2016). Cell-surface receptors include receptor-like kinases (RLKs) and receptor-like proteins (RLPs) that are able to detect pathogen-associated molecular patterns. Intracellular immune receptors detect specific effector molecules or their intermediates on host cell components (Andolfo and Ercolano 2015). These intracellular immune receptors, often encoded by *R* (resistance) genes, are modular proteins that typically carry a nucleotide-binding (NB) and a leucine-rich repeat domain (LRR) referred to also as NOD-like receptors (NLRs) (Jones et al*.* 2016).

NLR proteins are grouped into two classes: Toll/interleukin receptor (TIR)–NB–LRR proteins (TNLs) and non-TNL proteins. These two subfamilies are distinguished by the presence/absence of an N-terminal signaling domain, namely the TIR/resistance protein domain (McHale et al*.* 2006; Shao et al*.* 2016). Several non-TNL proteins possess a coiled coil (CC) motif at the N-terminus and are thus known as CC–NB–LRR proteins (CNLs) (Xiao et al*.* 2001). CNLs that contain an EDVID amino-acid motif (C_EDVID_NLs) or an RPW8-like protein (C_RPW8_NLs, CC_RPW8_-NB–LRR) were also found (Xiao et al*.* 2001; Shao et al*.* 2014). In addition, the identification of NLRs with integrated domains (IDs) that resemble pathogen targets may suggest a convergent functional evolution (Cesari et al*.* 2014; Sarris et al*.* 2016; Urbach and Ausubel 2017).

Recently, it was proposed that NLRs originated in green algae chlorophytes (Shao et al. 2019) but it remains unclear how the NB–LRR architecture was generated and scattered across diverging lineages. Genomic sequence comparisons across species have shown that proteins with the NB, LRR, and TIR domains are widespread and that gene structure and composition have been modified through time (Yue et al*.* 2012). Events such as gene fusion and fission play important roles in generating novel genes and functions, as they are the primary source of new domain architectures and biological innovation (Wu et al*.* 2012). Protein modifications occur primarily to alter the molecule conformation to modulate protein interactions, activity and stability (Bourne 1988; Müller 2018).

Eukaryotic algae evolved independently in different lineages (Blaby-Haas and Merchant 2019). Phylogenetic analyses have established that a primary endosymbiosis between a photosynthetic cyanobacterium and a colorless eukaryotic host gave rise to a plastid-harboring protist ancestor that, in turn, gave rise to Rhodophyta, Glaucophyta, Chlorophyta, and Streptophyta (Qiu et al*.* 2013; Jill Harrison 2017). Thus, the analysis of algal genomes offers a valuable opportunity to follow the modular organization assembled from a toolkit of domains such as NB, LRR, and TIR. Several studies on the defense responses of algae showed that they possess a cell surveillance system based on sensor and signal transduction components. The molecular basis of pathogen recognition in algae is strikingly similar to those found in animals and land plants, suggesting that the underlying biochemical machinery arose early in evolution (Potin et al*.* 2002). In addition, some unicellular algae, including chlorophytes, show characteristic apoptotic-like features (Segovia et al. 2003). The intimate contact occurring in parasitism, symbiosis, pathogen, epiphyte, and entophyte interactions could promote horizontal gene transfer (HGT), a major driver of genome evolution in bacteria and archaea, but more rare in eukaryote genomes (Gao et al*.* 2014; Soucy et al*.* 2015). However, in few cases do HGT events appear to be important for promoting genomic variation and biological innovation also in plants (Rinerson et al*.* 2016). To date, no HGT events involving protists have been reported for NLR loci. Such gaps in knowledge were the main drivers of our research.

In this study, we performed comparative genomic and phylogenetic analyses of NB-encoding genes with an emphasis on the basal-branching of Viridiplantae to follow domain reorganizations and to identify the first NB and LRR domain associations. Chlorophyte genomes were further investigated to evaluate the hypothesis that a horizontal transfer event induced the assembly of the basic NLR protein unit (NB–LRR). We looked into the structural and functional organization of the model green alga genome (*Chromochloriszofingiensis*) to obtain new insights into the evolution of immune receptors. Our findings provide an innovative evolutionary vision on the origin and diversification of LRR immune receptors in plants.

## Materials and Methods

### Taxa Data Set

To investigate the evolution of domain combinations of NLR genes along the plant life tree, we used the genome sequences of 41 taxa (24 bacteria, 1 archaeon, 1 glaucophyte, 1 phaeophyte, 2 rhodophytes, 8 chlorophytes, 1 charophyte, 1 liverwort, 1 moss, and 1 lycopodiophyte). The strain Chromochloris zofingiensis (Dönz) Fučíková et Lewis (ACUF 684) and Klebsormidium flaccidum (Kützing) Silva P.C., Mattox K.R. et Blackwell W.H. (ACUF 065) were in the algal collection of the Department of Biology, University Federico II of Naples (www.acuf.net). The analyzed proteomes were downloaded from Phytozome v11 (http://phytozome.jgi.doe.gov/) and other genome websites (supplementary table 1, Supplementary Material online). The differences in genome assembly can affect the number of NB genes identified. To reduce the risk of bias in NLR identification, gene annotations were conducted on the soft-masked versions of the most recent genome assemblies (Bayer et al*.* 2018). However, small inaccuracies could still be present in performed annotations. Moreover, for comparative purposes we also added 70 well-characterized cloned reference *R* genes identified in 20 tracheophyte species (vascular plants) retrieved from PRGdb (Osuna-Cruz et al*.* 2018) (supplementary table 2, Supplementary Material online). The selected organisms covered a broad diversity of taxonomic groups tracing the evolution of higher plants (supplementary table 3, Supplementary Material online).

### Identification of NB-Encoding Genes

To identify NB-encoding genes, we scanned the proteome data set (supplementary table 2, Supplementary Material online) with the hidden Markov model of the NB domain (Pfam: PF00931) using HMMER v3.0 with default parameters (Finn et al*.* 2011). The seed sequence of the NB domain was retrieved from Pfam v31.0 (http://pfam.xfam.org). In addition, a local BlastP search was performed by mapping *R*-gene motif sequences to our protein data set (*E* value cutoff of 10).

The protein domain architecture of HMMER and BLAST outputs was annotated using InterProScan (Jones et al*.* 2014) and conserved domain search (Marchler-Bauer et al*.* 2017) with default parameters.

BLASTp analyses, performed to identify the best hits and the *R*-gene homologs of green algae NLR-like genes, were implemented on the NCBI BLASTp website (http://blast.ncbi.nlm.nih.gov), using default settings (supplementary table 3, Supplementary Material online).

### Sequence Alignments

Multiple alignment using fast Fourier transform v6.814b (Katoh et al*.* 2002) was employed to align the NB Pfam domain of annotated proteins of NB-encoding genes, using the L-INS-i algorithm. Green algae full-length protein sequences were aligned with ClustalW (Larkin et al*.* 2007), using default settings.

Multiple alignment of conserved genomic sequence with rearrangements software package v2.2.0 (http://asap.ahabs.wisc.edu/mauve) was used to align homologous regions among two or more genome sequences. To determine a reasonable value for the minimum locally collinear block (LCB) weight, we performed an initial alignment with the default value and then used the LCB weight slider in the mauve GUI to eliminate all spurious rearrangements. The sequences were then realigned using the manually determined weight value.

### Phylogenetic Analysis

**Fig. 1.**
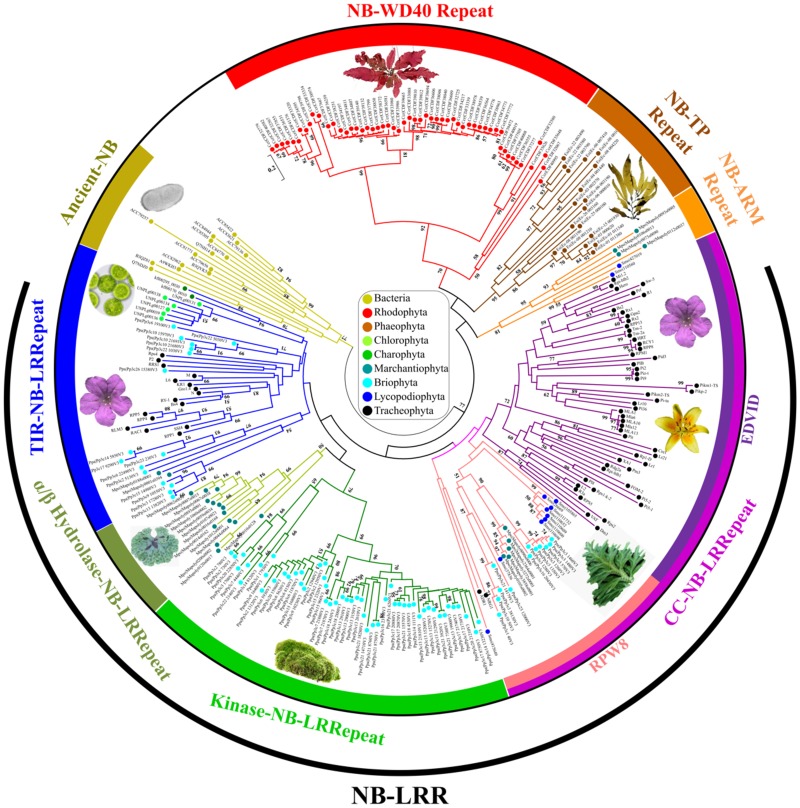
—Natural diversification of the NB-encoding gene families retrieved from bacteria, archaea, glaucophytes, algae, and bryophytes. The evolutionary history of 217 NB-encoding genes, harboring at least 50% of the NB Pfam domain, was used together with 70 well-characterized plant *R* genes to perform a maximum likelihood analysis. Labels showing the bootstrap values higher than 50 (out of 100) are indicated above the branches. The taxa to which the protein sequences belong are indicated by colored spots.

**Fig. 2.**
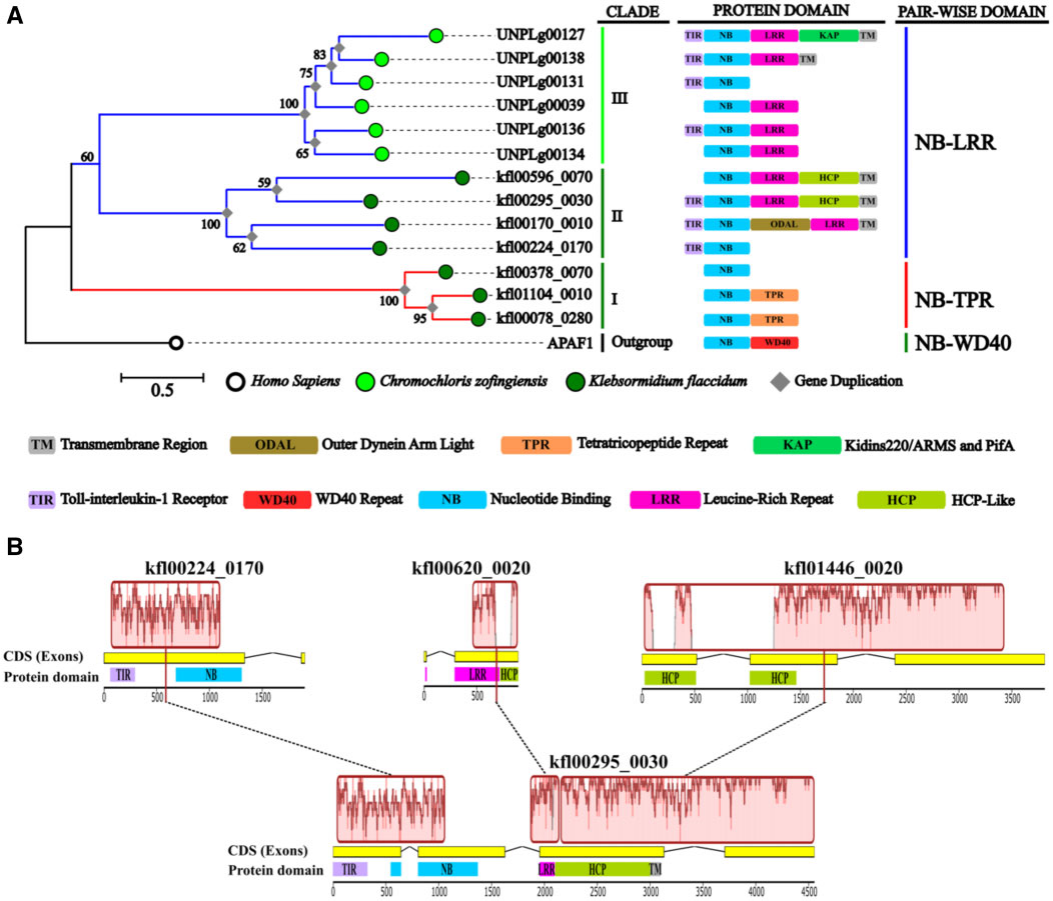
—Maximum likelihood phylogenetic tree and genomic reshuffles of green algae NB-encoding genes. (*A*) The tree includes 13 genes and one outgroup gene with an NB domain, the human *APAF1*. Clades were numbered with Roman numerals from I to III. Bootstrap values higher than 50 (out of 100 replicates) are indicated above the branches. (*B*) Identification of putative genomic reshuffles in a *Kf*NLR locus (KFL00295_0030) is shown. The LCBs (red boxes) are conserved segments, that appear to be internally free from gene rearrangements. Collinear blocks are connected by black-dotted lines, whereas block boundaries indicate breakpoints.

Evolutionary analyses were conducted using MEGA7 (Kumar et al*.* 2016). The phylogenetic relationships of annotated proteins from NB-encoding genes (fig. 1), green algae NLR-like proteins (fig. 2*A*), N-terminal regions (sequence upstream of the NB Pfam domain) of green algae NLR-like and plant R proteins, and C-terminal regions (LRR Superfamily domain: SSF52058) of green algae NLR-like proteins were inferred using the maximum likelihood method based on Jones et al*.* (1992) w/freq. model. The model with the lowest Bayesian information criterion score was considered to better describe the substitution pattern. The bootstrap consensus tree inferred from 100 replicates was taken to represent the evolutionary history of the sequences analyzed (Felsenstein 1985). The trees were drawn to scale, with branch lengths measured by estimating the number of substitutions per site. Duplication events of green algae NLR-like genes were inferred using the method described by Zmasek and Eddy (2001) and implemented in MEGA7.

### Evolutionary Divergence Estimation

The average evolutionary divergence was separately calculated on the N-terminal region upstream of the predicted NB Pfam domain of 53 CNL R proteins, 14 TNL R proteins, and 8 green algae TNL-like protein sequences. Analyses were conducted using the JTT matrix-based model (Jones et al*.* 1992). All positions with less than 5% site coverage were eliminated. Evolutionary analyses were conducted in MEGA7 (Kumar et al*.* 2016).

### De Novo Prediction of NB Encoding Gene Motifs

The multiple EM for motif elicitation (MEME) (http://meme-suite.org/) algorithm (Bailey et al*.* 2009) was used to identify motifs (supplementary table 4, Supplementary Material online) in the NB Pfam domain (PF00931) of nucleotide binding tetratricopeptide repeat (NB-TPR) genes grouped in clade I (fig. 2*A*). The analysis was carried out using the default cutoff value for statistical confidence. The Motif Alignment and Search Tool (http://meme-suite.org/) was also used to confirm the presence of MEME-motifs previously identified (supplementary table 4, Supplementary Material online), using default settings.

### Detection of Codon Usage Bias

The synonymous codon usage bias of *C. zofingiensis* NLR-like (*Cz*NLR) genes was determined with the web-application E-CAI server (http://genomes.urv.es/CAIcal/E-CAI). The codon adaptation index (CAI) value was calculated to measure the synonymous codon usage bias. CAI values range from 0 to 1, being 1 if a gene always uses the most frequently used codon of a reference set (Puigbò et al*.* 2008). Comparisons of codon usage preferences were performed against reference sets from *C. zofingiensis* (codon usage table of nuclear coding DNA sequences, CDSs) (supplementary table 5, Supplementary Material online) using the standard genetic code.

To evaluate the statistical support of the CAI values, we defined a threshold value or expected-codon adaptation index (e-CAI) by generating random sequences with Guanine–Cytosine (GC) content, amino acid (AA) composition and sequence length similar to the query sequences (*Cz*NLR genes). CAI values above the e-CAI were interpreted as statistically significant, meaning that codon similarity arose from codon preferences rather than from internal biases (Puigbò et al*.* 2008).

### GC Content Profile

GC content profile along the Cz1030-34550 region of the *C. zofingiensis* Un55705 chromosome was characterized using the GC‐Profile webserver (http://tubic.tju.edu.cn/GC-Profile/) with recommended values for halting parameter (100) and minimum segment length (2,000 bp).

### Detection of Horizontal Gene Transfer Events

To detect candidate HGT events, an alien index (AI) was calculated as described by Gladyshev et al*.* (2008) and Flot et al*.* (2013). All *Cz*NLR proteins were compared with NCBI’s nonredundant protein library using BLASTp, with kingdom and taxon ID assignment, and an *E* value threshold of 1e^−5^. An AI could only be calculated for a protein returning at least one hit in either Viridiplantae or non-Viridiplantae species, as stated in the following formula: AI = log ((Best *E* value for Viridiplantae) + e^−200^) – log ((Best *E* value for non-Viridiplantae) + e^−200^).

When BLASTp results were not identified for either Viridiplantae or non-Viridiplantae, the query sequence (NLR-like proteins) was removed from downstream analysis. BLASTp results in the phylum Chlorophyta (to which *C. zofingiensis* belongs) were ignored for the calculation of AI to allow the detection of putative HGT events that could be shared with other related species.

An AI > 30 corresponded to a difference of magnitude e10 between the best non-Viridiplantae and best Viridiplantae *E* values and it was estimated to be indicative of a potential HGT event (Flot et al*.* 2013). Sequences with an AI > 30 and >70% identity to a non-Viridiplantae sequence were considered putative contaminants and removed from further analyses. The HGT prediction tool set is available at Github: https://github.com/peterthorpe5/public_scripts/tree/master/Lateral_gene_transfer_prediction_tool.

### Transcriptional Validation of *Cz*NLR Genes

Nucleic acids (gDNA and RNA) were obtained from cultures of *C. zofingiensis* grown in 100 ml of basal bold medium (Stein 1973) at 24 °C with a photoperiod of 16 h light and 8 h dark. *Chromochloriszofingiensis* culture with an optical density of 1.0 (λ 600 nm) was left in the dark for 48 h. The growth condition (48-h darkness) for *Cz*NLR-operon validation was selected on the basis of the FPKM-profile reported by Roth et al. (2017) (supplementary table 6, Supplementary Material online). Cells were collected by centrifugation at 4,000 rpm for 15 min at 4 °C and the pellet was rinsed twice with cold sterile MilliQ water (Millipore). The *C. zofingiensis* biomass was rapidly frozen by immersion in liquid N_2_.

Total RNA was isolated from finely ground, frozen *C. zofingiensis* biomass using the Spectrum Plant Total RNA Kit (Sigma-Aldrich). Complete removal of traces of DNA was performed using On-Column DNase I Digest Set (Sigma-Aldrich). Reverse transcription was performed using the SuperScript III Reverse Transcriptase kit (Thermo Fisher Scientific) with oligo-dT primers. cDNAs were diluted (1:5) with autoclaved distilled water and stored at −20 °C until further analysis. Genomic DNA (gDNA) was obtained using the DNeasy Plant Mini Kit (Qiagen). gDNA and RNA quantities were determined by the NanoDrop ND-1000 Spectrophotometer (NanoDrop Technologies).

To evaluate the expression of NLR-like genes in *C. zofingiensis* a PCR analysis was carried out on diluted cDNAs using Takara LA Taq DNA polymerase (Cat. No. RR002A). Reactions were prepared in a total volume of 25 µl and 0.2 µM pmol of target gene primers (supplementary table 7, Supplementary Material online) and 1 µl of cDNA template. PCR cycling conditions were as follows: 94 °C for 1 min, followed by 30 cycles of two steps: 98 °C for 10 s and 68 °C for 15 min followed by a single cycle of 68 °C for 8 min. Primers designed on the housekeeping gene Cz05g19160 locus (*ACT*) or a portion of actinA mRNA sequence (supplementary table 7, Supplementary Material online) were used as control reaction (Lee et al*.* 2015). For each primer set, the control reactions, in which reverse transcriptase (but not DNA polymerase) was omitted, showed that the product amplification was not due to DNA contamination. Two specific couple of primers were tested on a gene–gene junction of the OPERON-2 genomic region (supplementary table 7, Supplementary Material online).

### Prediction of α-Transmembrane Regions

Transmembrane (TM) domain (amino acidic region of single alpha helix) detection of green algae NLR-like proteins was implemented in PHOBIUS (http://phobius.sbc.su.se/), a transmembrane topology and signal peptide predictor. To verify the putative TM-domains, we investigated the green algae NLR-like proteins with six TM-predictors, based on physicochemical (DAS-TM: http://www.enzim.hu/DAS/DAS.html; PRED-TMR: http://athina.biol.uoa.gr/PRED-TMR/input.html), statistical (TMPRED: https://embnet.vital-it.ch/software/TMPRED_form.html; SPLIT: http://splitbioinf.pmfst.hr/split/4/), and machine learning (TMHMM2: http://www.cbs.dtu.dk/services/TMHMM/; TOPCONS: http://topcons.cbr.su.se/) methods (Venko et al*.* 2017).

## Results

### Tracing the NLR Evolution Routes

The evolutionary events responsible for the genesis of the NLR proteins were inferred by analyzing the NB domain rearrangements along the plant tree of life. The NB association paths were traced by comparing over 700 NB protein-encoding genes belonging to 41 taxa, including prokaryotic genomes (supplementary tables 2 and 8, Supplementary Material online), because a common bacterial ancestor gave rise to the diversification of the metazoan NACHT and the plant NB domains (Leipe et al*.* 2004). For comparative purposes 70 well-characterized plant *R* genes were also added to our data set (supplementary table 9, Supplementary Material online). The evolutionary architecture of NB-encoding genes retrieved in bacteria, archaea, glaucophyte, algae, and bryophyte lineages, revealed a clear distinction among ancient-NB, NB–LRR, NB-armadillo repeat, NB-TPR, and nucleotide binding WD40 repeat (NB-WD40) (fig. 1).

Despite prokaryotic NB-encoding genes showed a modular domain organization (TIR–NB, NB-WD40, and NB-TPR) similar to other analyzed organisms, they clustered separately in the generated phylogenetic tree (gold clade in fig. 1). Only 9 out of 25 analyzed prokaryote genomes included NB-encoding genes. Over 40% (13 out of 31) of identified NB-encoding genes in bacteria genomes presented a TPR domain (Pfam-IDs: PF13424) and ∼35% (11 out of 31) were associated with a WD40 domain (Pfam ID: PF00400). The LRR domain (Pfam ID: PF13855) was found alone or associated with other domains in *Methanosarcina mazei* (Archaeon). The first domain associations similar to the *R* genes (TIR–NB and TIR–LRR) were observed in two bacteria species (*Chloroherpeton thalassium* and *Rhodopirellula baltica*).

Moving within the Rhodophyta algae phylum (red clade in fig. 1), the pluricellular red alga *Chondrus crispus* revealed a total of 61 NB-encoding genes of which 95% (58 out of 61) showed a NB-WD40 domain association and many of them (23 out of 58) included a TIR domain as well as a transmembrane Dipeptidyl Peptidase-like Protein 6 domain involved in membrane trafficking (Lin et al*.* 2014). In addition, three *C. crispus* NB-WD40 proteins displayed a YVTN repeat-like domain (IPR011044), typical of archaeal surface layer proteins (PMID: 12377130), and five *C. crispus* NB-encoding genes contained a TBPIP domain (Pfam ID: PF07106) involved in viral interaction (Ijichi et al*.* 2000). In contrast, *Galdieria sulphuraria*, an extremophilic unicellular species, included only five LRR-encoding genes. The great majority of NB proteins revealed in brown algae genomes contained a TPR domain at the C-terminal and a transmembrane signature (brown clade in fig. 1).

Different NB domain arrangements were found in the chlorophyte lineage. Notably, domain reshuffling was observed in the unicellular green alga *C. zofingiensis* which showed the most ancestral NLR-like genes. Moreover, seven NB-encoding genes including atypical NLR assemblies were found in *Klebsormidiumflaccidum*, a charophyte multicellular green alga more closely related to land plants. Several TNL and C_RPW8_NL were identified in nonflowering plants (common liverwort: *Marchanthia polymorpha*; moss: *Physcomitrella patens*) (blue and pink clades in fig. 1 and supplementary fig. 1, Supplementary Material online). Over 350 NLR-like genes, including the kinase domain (Pfam ID: PF00069) (green clade in fig. 1), TPR domain short residues, ZZ zinc finger domain (Pfam ID: PF00569), and the LAZ5 domain (PANTHER ID: PTHR11017: SF181), were observed in *P. patens* proteins. NB-encoding genes with an α/β hydrolase N-terminal domain were observed in *M. polymorpha* (dark green clade fig. 1) and NB proteins with the ARM domain (Superfamily ID: SSF48371) were found in *Selaginella moellendorffii* (lycophyte).

### Back to the Origin of the Basic *R*-Gene Unit (NLR)

A detailed phylogenetic analysis performed on the green algae *K. flaccidum* and *C. zofingiensis* NB-encoding genes allowed three distinct branches to be visualized (fig. 2*A*). Clade I included *K. flaccidum* genes with NB-TPR association (red in fig. 2*A*). The NB Pfam domain (PF00931) of these *K. flaccidum* members lacked the typical R protein signatures (PRINTS ID: PR00364) and displayed other specific motifs (supplementary table 4, Supplementary Material online). The *K. flaccidum* NLR-like (*Kf*NLR) genes, which showed a relatively high homology with the TIR–NB region of the plant R proteins, were grouped in clade II (fig. 2*B*;supplementary table 2 and fig. 2, Supplementary Material online). These observations suggested that a divergent evolution occurred in the formation of the NB domain of members of clades I and II. In contrast, *Cz*NLR proteins grouped in clade III showed a domain composition similar to the *R* genes of flowering plants. Inference analysis of green alga NLR-like genes revealed that fusion, fission, and duplication events (gray diamonds in fig. 2*A*) have considerably shaped their structure. The architecture of *Kf*NLRs (clade II) suggested that an event of gene-fusion occurred between NB-encoding genes and Sel1-like repeats (SLRs), adaptors for assembly macromolecular complexes found in bacterial proteins or in bacterial virulence factors, including an outer dynein arm light (ODAL) domain (Superfamily ID: SSF52075) showing LRR motifs.

### Horizontal Transfer Promoted the Alien LRR Domain Acquisition

The presence of bacteria-like domains (HTH cro/C1-type domain-containing protein, NEL, novel E3 ligase; PSA, parasite surface antigen glycoprotein) in the architecture of *Kf*NLR genes encouraged us to investigate whether these domains were acquired by HGT events. To verify the full/partial transfer of NLR-encoding regions, we analyzed their codon usage distribution, GC content and the percent of identity to other species.

**Fig. 3.**
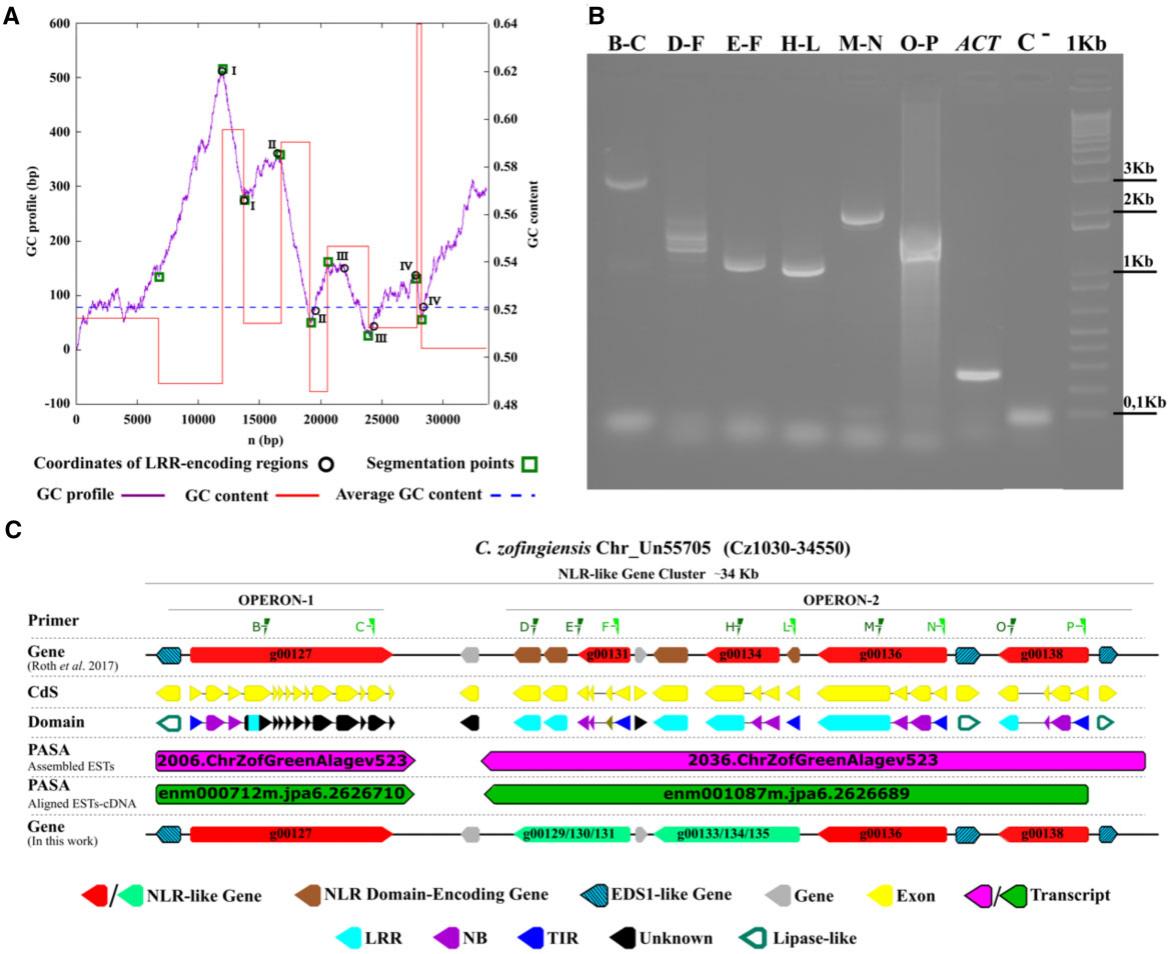
—Prediction and horizontally transferred genes and genomic characterization of the two functioning units of *C. zofingiensis* genomic DNA (operons). (*A*) The negative cumulative GC-profile (violet line) for the Cz1030-34550 region of *C. zofingiensis* Un55705 chromosome (corresponding to the NLR-like gene cluster reported in panel *C*), marked with the segmentation points (green square) revealed. (*B*) Gel shows the genetic transcription of *Cz*NLRs and the molecular validation of two erroneously split *Cz*NLR genes (g00129/g00130/g00131 and g00133/g00134/g00135). The primer pairs (green triangles) designed in the Cz1030-34640 region are shown at the top. Control RT-PCRs amplifying a section of the Cz05g19160 locus (ACT) or a portion of the actinA mRNA sequence are also shown (Lee *et al.* 2015). (*C*) A cluster of NLR genes (red arrows) containing the two operons located on the Cz1030-34550 region of chromosome Un55705.

To verify the codon usage similarity of each *Cz*NLR–CDSs with the rest of the *C. zofingiensis* nuclear gene complement, we performed an e-CAI analysis. The *Cz*NLR–CDSs showed a CAI value which was significantly different from the *C. zofingiensis* e-CAI (table 1). This finding supported a *Cz*NLR atypical nucleotide composition and a putative horizontal transmission (table 1). A strong deviation of GC content from the genomic average GC signature was observed in the LRR-coding regions of four *Cz*NLR genes (fig. 3*A*). These DNA segments had a base composition that diverged significantly from the overall base composition of the chromosome UN55705 (fig. 3*A*), suggesting that a DNA insertion from a distantly related organism had occurred (Ravenhall et al*.* 2015). Finally, an AI analysis, based on *e* values obtained from BLAST against the NCBI’s nonredundant protein library, confirmed the acquisition of LRR-regions (except for UNPLg00127, probably because of a short LRR-region) by HGT of Kinetoplastida-origin in *C. zofingiensis* (table 2).

**Table 1:** CAI Values for *Cz*NLR–CDSs Compared against the *Chromochloris zofingiensis* Nuclear Genome Codon Usage Table

Gene ID^a^	CAI (average)	e-CAI^b^ (*α* = 0.05)	Statistical Tests (*α* = 0.05)	
			K–S^c^	*χ* ^2^ (confidence level)^d^ (%)
UNPLg00136	0.606	0.664	0.032	100
UNPLg00133/134/135	0.621	0.683	0.028	100
UNPLg00039	0.614	0.676	0.034	100
UNPLg00138	0.616	0.678	0.032	100
UNPLg00127	0.614	0.674	0.030	100
UNPLg00129/130/131	0.611	0.675	0.021	100

Note.—For each *Cz*NLR–CDS the CAI value, e-CAI value (determined by randomly generating 500 sequences with the same GC content and AA composition of *Cz*NLR–CDSs) and statistical parameters (chi-square, *χ*^2^ goodness-of-fit test and Kolmogorov–Smirnov, K–S test) are reported.

a With respect to the new NLR gene annotation performed in this study (see fig. 3*C*).

b e-CAI is a threshold value for discerning whether the differences in the CAI value are statistically significant. The e-CAI value was calculated at a 95% level of confidence using the Markov method.

c K–S test confirmed whether the CAI of the randomly generated sequences follow a normal distribution. K–S test values for each calculated e-CAI is always lower than of critical value (0.061).

d A *χ*^2^ test is conducted to compare the goodness-of-fit between the AA frequencies or GC content of each *Cz*NLR–CDS and their mean values.

**Table 2:** List of HGT Events Occurring in the *Cz*NLR Gene Family

Gene ID^a^	AI Category^b^	AI	BLAST *e* Value	% Identity	Taxon
UNPLg00136	Very likely HGT	161.2	2.0E-126	40.4	Kinetoplastida
UNPLg00133/134/135	Very likely HGT	82.9	2.0E-89	45.0	Kinetoplastida
UNPLg00039	Very likely HGT	108.9	1.0E-105	43.0	Kinetoplastida
UNPLg00138	Possible HGT	23.6	5.0E-28	37.8	Kinetoplastida
UNPLg00129/130/131	Very likely HGT	105.9	3.0E-104	42.1	Kinetoplastida

Note.—For each gene the AI category, the AI value, the BLAST *e* value, the percentage of identity (% identity), and the taxonomic group of the candidate donor are indicated.

a In respect of new NLR gene annotations performed in this study (see fig. 3*C*).

b BLAST results are classified in two categories: very likely HGT (AI > 30 and <70% identity to candidate donor) and possible HGT (AI > 0 and <70% identity to candidate donor).

The genomic organization of *Cz*NLRs supported the hypothesis that an HGT event lay at the basis of NLR unit origin. Indeed, a fine gene annotation of the Cz1030-34550 region (∼34 kb) on UN55705-chromosome showed that five *Cz*NLR genes (red in fig. 3*C*) clustered in an operon-like structure (fig. 3*C*). The six distinct genes (g00129, g00130, g00131, g00133, g00134, and g00135), incorrectly divided in the official annotation (Roth et al. 2017), were grouped into two *Cz*NLR genes (green in fig. 3*C*). In silico analyses showed that the operon-like structures are transcribed together into two mRNA strands. On the basis of domain characterization, gene expression profile, and molecular validation, a new NLR annotation was proposed (light green arrows) (fig. 3*B*). It is also worth noting that in this region we identified three enhanced disease susceptibility 1 genes, positive regulators of innate immunity mediated by TNL resistance proteins (Bhattacharjee et al*.* 2011) (striped cyan arrows in fig. 3*C*). Transcriptome data reported by Roth et al. (2017) suggest that operon genes are transcribed together into an mRNA strand (violet and green arrows in fig. 3*C*). Molecular analysis carried out in this study confirmed the transcription of operon-like structures into two polycistronic mRNAs (supplementary fig. 3 and table 10, Supplementary Material online).

### Subcellular Localization of the NLR Proteins Progenitor

**Fig. 4.**
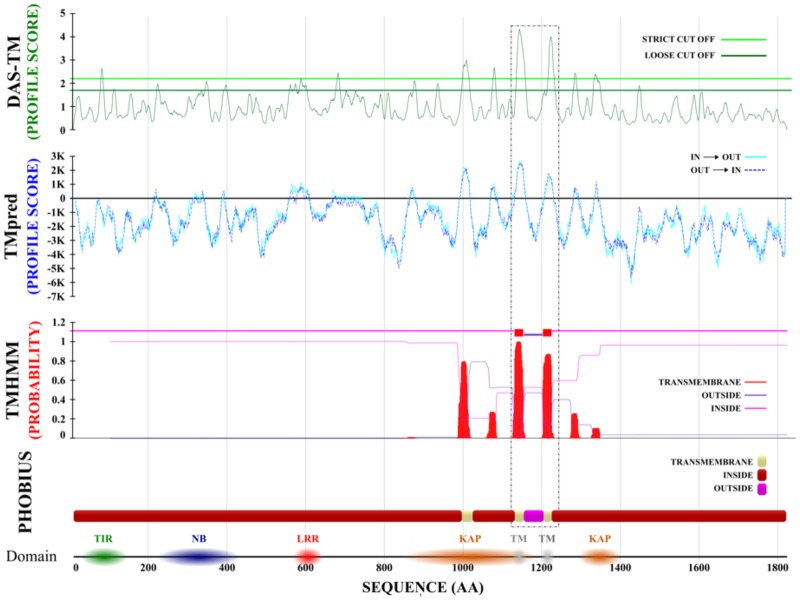
—In silico validation of PHOBIUS α-TM regions in the *C. zofingiensis* UNPLg00127 locus. From top to bottom, we report the putative TM-region predictions of DAS-TM, TMpred, and TMHMM software (Venko *et al.* 2017). A dashed black box underlines two PHOBIUS α-TM regions (ivory boxes), which were confirmed by all the predictors used (green, blue, and red peaks of DAS-TM, TMpred, and TMHMM predictions, respectively).

PHOBIUS prediction software detected several putative α-transmembrane (α-TM) regions in five *C. zofingiensis* green-algae NLR-like proteins (fig. 2*A*). To further investigate the matter, we performed additional analyses with six TM-predictors, based on physicochemical, statistical, and machine learning methods (Venko et al*.* 2017). Comparative prediction confirmed the presence of at least one α-TM region for each analyzed NLR-like gene, suggesting cellular membrane localization of R proteins progenitors (supplementary table 11, Supplementary Material online). Figure 4 reports the prediction of two highly probable α-TM encoding regions in *C. zofingiensis* UNPLg00127 locus. Protein domain scanning identified the presence of a Kidins220/ARMS/PifA-NTPase domain at the C-terminal including two/four transmembrane helices to anchor proteins to the membrane (Aravind et al*.* 2004) that supported its putative TM-localization. Inference analysis on the C-terminal regions (LRR Superfamily domain: SSF52058) of green algae NLR genes underlined a greater homology (bootstrap index = 90) with the extracellular-LRR domain of transmembrane receptors RLP and RLK (supplementary fig. 4 and table 3, Supplementary Material online) and with the intracellular-LRR domain of Arabidopsis *TAO1* gene, a peripheral plasma membrane gene that confers resistance to the effector AvrB of *Pseudomonas syringae* (supplementary table 3, Supplementary Material online).

## Discussion

The multitaxa investigation of NB-encoding genes performed in this study allowed us to discover the evolutionary dynamics that generated plant *R* genes. Taxon-specific rearrangement in bacteria, fungi, plants, and animals originated, using a finite library of domains, assorted NB proteins with different physical structures and functional activity (Sanseverino and Ercolano 2012; Dyrka et al*.* 2014; Jones et al*.* 2016). The evolutionary path from ancestral NB-encoding genes to plant NLRs likely occurred via bacterial intermediaries, such as TIR–NB- or TIR–LRR-encoding genes (Urbach and Ausubel 2017). In bacteria and algae NB was preferentially associated with a WD40 or TPR domain with or without transmembrane signatures. NB-WD40 and NB-TPR proteins carry out essential roles in many organisms, ranging from signal transduction to apoptosis (Shakeri et al*.* 2017; Heller et al*.* 2018). Such domain arrangements prove to be crucial in different recognition/transduction events linked to responses to environmental stimuli (Grove et al*.* 2008; Gao and Stock 2009; Zhou et al*.* 2018). The first NLR-domain associations found in algae chlorophyte lineage date back over 3.5 billion years ago. Independent NLR-domain associations have been found in Chlorophyta algae and in Charophyta freshwater algae, a paraphyletic group belonging to the Streptophyta lineage (Sarris et al*.* 2016; Shao et al*.* 2016; Gao et al*.* 2018), indicating that convergent evolution may have generated similar protein structures in separate lineages.

Combinatorial assembly of domains from nonhomologous proteins and/or exchange of smaller polypeptide segments defined the architecture of green plant NB-encoding genes (Riechmann and Winter 2000). An intense reassembly activity of NLR genes took place in primitive nonvascular plants under the guidance of a scaffold, following a structural transition and a series of smaller nondeleterious changes (Bornberg-Bauer et al*.* 2010). In bryophytes (∼400 million years ago) a strong expansion of the NLR family consolidated the NB–LRR domain association and promoted reshuffling at the N- and C-terminal regions respectively, enhancing the functional specialization and the variability of proteins (Sarris et al*.* 2016; Ortiz and Dodds 2018). The differences in genome assembly of the species analyzed may influence the number of duplicated genes identified because the NLR genes can collapse in genome assemblies (Bayer et al*.* 2018). In future, the magnitude of bryophyte NLR expansions may require some adjustments, but the variability observed at the N-terminal in *P.patens* (kinase-NLR) and in *M.polymorpha* (α/β Hydrolases-NLR) is well supported by previous findings (Ortiz and Dodds 2018).

After the events described above, the NLR pairwise domain combination became the “Supra-Domain” of the plant *R*-gene family. Despite the plethora of NB domain associations found across taxa, the NB–LRR domains unit was typical of green plants. Indeed, the NLR-family of green plants was characterized by a pairwise domain combination (NB and LRR) which was highly duplicated and frequently linked to other partner domains (TIR, α/β Hydrolases, Kinase, RPW8, CC, and ID). Events occurring at sublocus level, such as fusion and/or fission, may have played an important role in shaping the gene modular structure (Wu et al*.* 2012), although the repertoire of immune receptor domain combinations is not random in nature (Sanseverino and Ercolano 2012). The consolidation of NB–LRR coupling paved the way for the functional specialization of plant *R* genes, enforcing evolutionary paths that preserve the protein structure, despite the high sequence divergence of single terminal domains (Gilson et al*.* 2017). Several studies indicate that NLRs may have integrated new domains independently and frequently at various locations in their architecture during evolution (Han 2019). The functional properties of new proteins led to the selection of the most advantageous NB–LRR domain association. Indeed, NB–LRR architecture can easily evolve new binding specificities under diversifying selection, without sacrificing the stability of resistance supra-domain (R-SD) (Urbach and Ausubel 2017).

The discovery of bacteria-derived domains (HCP, NEL, and PSA) in the architecture of green algae NLR-like genes suggests that horizontal transfer events contributed to the origin of the plant NLR gene family. In eukaryotes, HGTs appear to be rare, except for parasitic plants that enrich through HGTs their host plants with defense proteins, including at least one NLR (Yang et al*.* 2016). Urbach and Ausubel (2017) and Han (2019) hypothesized a plausible acquisition by HGT of the NB domain from a different kingdom. To verify the full/partial transmission of *Cz*NLR-encoding regions from Protista, we analyzed their codon usage distribution, GC content and the percent of identity against other species. e-CAI analysis and GC-content performed in *Cz*NLR–CDSs revealed a nucleotide composition that significantly diverged from the overall base composition of the entire chromosome, suggesting that a DNA insertion from a distantly related organism occurred (Ravenhall et al*.* 2015). AI analysis confirmed the acquisition of LRR-regions by HGT of Kinetoplastida-origin in *C. zofingiensis*.

The Kinetoplastida is a group of protists involved in various lateral gene transfer events with algal- and α-proteobacterial-like endosymbionts (Hannaert et al*.* 2003). Dunin-Horkawicz et al*.* (2014) hypothesized that the eukaryotic innate immunity networks originated from their endosymbionts and that network complexity increased with the emergence of multicellularity. Interestingly, all alien domains found in this study are involved in pathogen–host recognition. In particular, the HCP motif of SLR bacterial proteins is involved in the signal transduction pathways during the interaction between bacterial and eukaryotic host cells (Mittl and Schneider-Brachert 2007). The ODAL domain (Superfamily ID: SSF52075) is highly homologous to the NEL domain (PFAM ID: PF14496) found at the C-terminus of bacterial virulence factors (Quezada et al*.* 2009; Singer et al*.* 2013). Likewise, the LRR domain (Superfamily ID: SSF5205) of *Cz*NLR was homologous to PSAs that present a Thr-/Ser-rich site implicated in defense mechanism (Lincoln et al*.* 2004).

HGT events in *Cz*NLRs further supported that a convergent evolution for these genes may have occurred. The identification of α-TM-regions (Cosson et al*.* 2013) in the protein architecture of NLR gene progenitors and a relatively high homology of the LRR-region to extracellular RLK and RLP receptors underpinned a putative cell-surface localization. Convergent evolution may have originated homologous proteins with slightly different domains to perform similar biochemical functions in Chlorophyta and Streptophyta. A *Cz*NLR membrane localization in unicellular and filamentous algae without differentiated or specialized cells could be important to perform a putative surveillance activity. It is also plausible that the subcellular translocation from plasma-membrane (green algae NLR-like genes) to cytoplasm (*R* genes) originated best suited proteins for the multilayered structure of the plant immunity network (Jones and Dangl 2006; Kamoun et al*.* 2018).

## Conclusions

The evolutionary divergence of NB-encoding proteins proved to be mediated by dynamic structural transition paths from an initial set of modular proteins. Cross species domain assemblies originated the first NLR genes identified in unicellular green algae. In silico transmembrane localization of ancient NLR genes is compatible with a “cell-surface surveillance” function. Moreover, the operon-like organization of such NLR members suggests that the plant *R*-gene clusters could derive from functional units. Evolution of the sophisticated NLR proteins occurring in Viridiplantae is based on the selection of an R-SD. The “pairwise unit” (NB–LRR) of R proteins evolves through reshuffles with subsets of protein domains that define specific protein structure and function in plant immunity.

### Ethics Approval and Consent to Participate

Not applicable. This manuscript does not report on or involve the use of any animal or human data or tissue.

### Consent for Publication

All of the authors read and approved the final manuscript.

### Availability of Data and Materials

All the supporting data are included within the article. The supplementary material (supplementary tables 1–11 and figs. 1–4, Supplementary Material online) for this article can be found online at: https://figshare.com/s/342d2d717961d3b537da. Users can download and use the data freely for research purposes only with acknowledgment to us and quoting this paper as reference to the data.

## Supplementary Material

Supplementary data are available at *Genome Biology and Evolution* online.

## Supplementary Material

evz248_Supplementary_Data
