# Supplementary material for: Alien Domains Shaped the Modular Structure of Plant NLR Proteins
Source: Genome Biol Evol. 2019 Nov 15;11(12):3466–77. doi: 10.1093/gbe/evz248 (PMC7145615; doi:10.1093/gbe/evz248)
Supplement: evz248_Supplementary_Data [file evz248_supplementary_data.zip › Andolfo_R2_Legends_Supplementary-materials.docx]

**Supplementary material:**

**Supplementary Figure 1**

**.ppt**

Phylogenetic tree of NB-encoding genes grouped in the pink and violet clades are reported in Fig. 1. Evolutionary history of 27 NB-encoding genes, harboring at least 50 % of the NB Pfam domain, were used together with 54 well characterized plant R-genes (2 C_RPW8_NL and 52 C_EDVID_NLs) to perform a maximum likelihood analysis. The taxa to which the protein sequences belong are indicated by colored spots. Finally, the different color branches denote the divergent clades associated to specific domain associations (CC_RPW8_-NB-LRR in pink, CC_EDVID_-NB-LRR in violet).

**Supplementary Figure 2**

**.ppt**

Maximum likelihood analysis of N-terminal regions upstream of the predicted NB Pfam domains of 8 green algae NLR-like proteins. For comparative purposes we used 67 well characterized plant *R*-genes (14 TNLs and 53 CNLs). The protein classes to which the R protein sequences belong are indicated by colored spots (TNLs in blue and CNLs in red). Green algae NLR-like proteins were indicated by light green spots.

**Supplementary Figure 3**

**.ppt**

Molecular validation to confirm the transcription of operon-like structures is shown. A) Gel shows the genetic transcription of OPERON-1 and OPERON-2 into two polycistronic mRNAs. B) Additional molecular validation based on primers spanning the two genes simultaneously. C) Genomic region containing the two operon-like structures. They are located on the Cz1030-34550 region of chromosome Un55705.

**Supplementary Figure 4**

**.ppt**

Inference analysis of C-terminal regions (LRR domains) of seven green algae NLR-like proteins, used together with 82 well characterized plant R-genes (11 RLPs, 7 RLKs, 15 TLNs and 49 CNLs) to perform a maximum likelihood analysis. The protein classes to which the reference protein sequences belong are indicated by colored spots (RLPs in red, RLKs in black, TNLs in blue and CNLs in dark green). Green algae NLR-like proteins were indicated by light green spots.

**Supplementary Table 1**

**.xlsx**

List of genome websites.

**Supplementary Table 2**

**.xlsx**

List of 41 sequenced genomes used in this study.

**Supplementary Table 3**

**.xlsx**

Functional characterization of green algae NLRs. The best-hits were identified using NCBI-BLASTp tool (default-setting). The protein query region (Full-length protein, TIR-NB region and LRR region), NCBI-Databases (nr and SwissProt), best-hit (NB-domain), hits and E-value are reported for each green algae NLR.

**Supplementary Table 4**

**.xlsx**

List of motifs identified in the NB Pfam domain (PF00931) of *K. flaccidum* NB-encoding genes belonging to the phylogenetic clade I (see Fig. 2A). For each gene, the motif sequence, p-value, the motif coordinates and the corresponding protein region are reported.

**Supplementary Table 5**

**.xlsx**

Codon usage table of nuclear coding DNA sequences identified in *C. zofingiensis* genome sequence.

**Supplementary Table 6**

**.xlsx**

FPKM-matrices of RNA-Seq analysis available as Datasets S20 reported by Roth *et al.,* (2017).

**Supplementary Table 7**

**.xlsx**

Gene-specific primers used in this study for gene-expression analysis.

**Supplementary Table 8**

**.xlsx**

List of NB-encoding genes identified in this study.

**Supplementary Table 9**

**.xlsx**

List of 70 well-characterized cloned reference R-genes.

**Supplementary Table 10**

**.xlsx**

Gene-specific primers used in this study for operon-like structures validation.

**Supplementary Table 11**

**.xlsx**

Comparison of six predictor tools employed for the identification of α-transmembrane regions in four green algae NLR-like proteins. We report the success or failure of α-transmembrane predictions.
