# Supplementary material for: Alien Domains Shaped the Modular Structure of Plant NLR Proteins
Source: Genome Biol Evol. 2019 Nov 15;11(12):3466–77. doi: 10.1093/gbe/evz248 (PMC7145615; doi:10.1093/gbe/evz248)
Supplement: evz248_Supplementary_Data [file evz248_supplementary_data.zip › Supplementary Figures_5Novembre2019.pptx]

## Slide 1
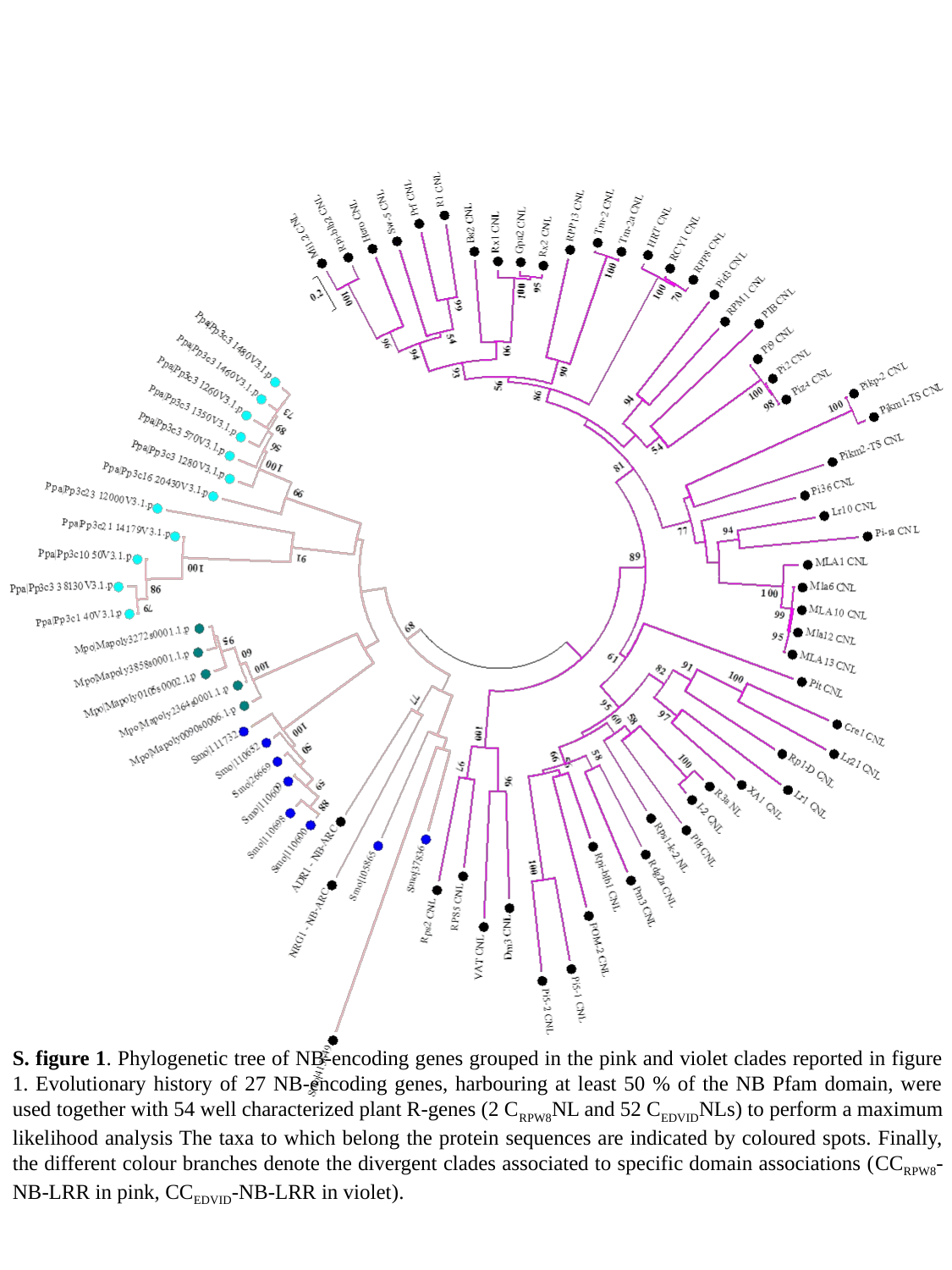

S. figure 1. Phylogenetic tree of NB-encoding genes grouped in the pink and violet clades reported in figure 1. Evolutionary history of 27 NB-encoding genes, harbouring at least 50 % of the NB Pfam domain, were used together with 54 well characterized plant R-genes (2 CRPW8NL and 52 CEDVIDNLs) to perform a maximum likelihood analysis The taxa to which belong the protein sequences are indicated by coloured spots. Finally, the different colour branches denote the divergent clades associated to specific domain associations (CCRPW8-NB-LRR in pink, CCEDVID-NB-LRR in violet).

## Slide 2
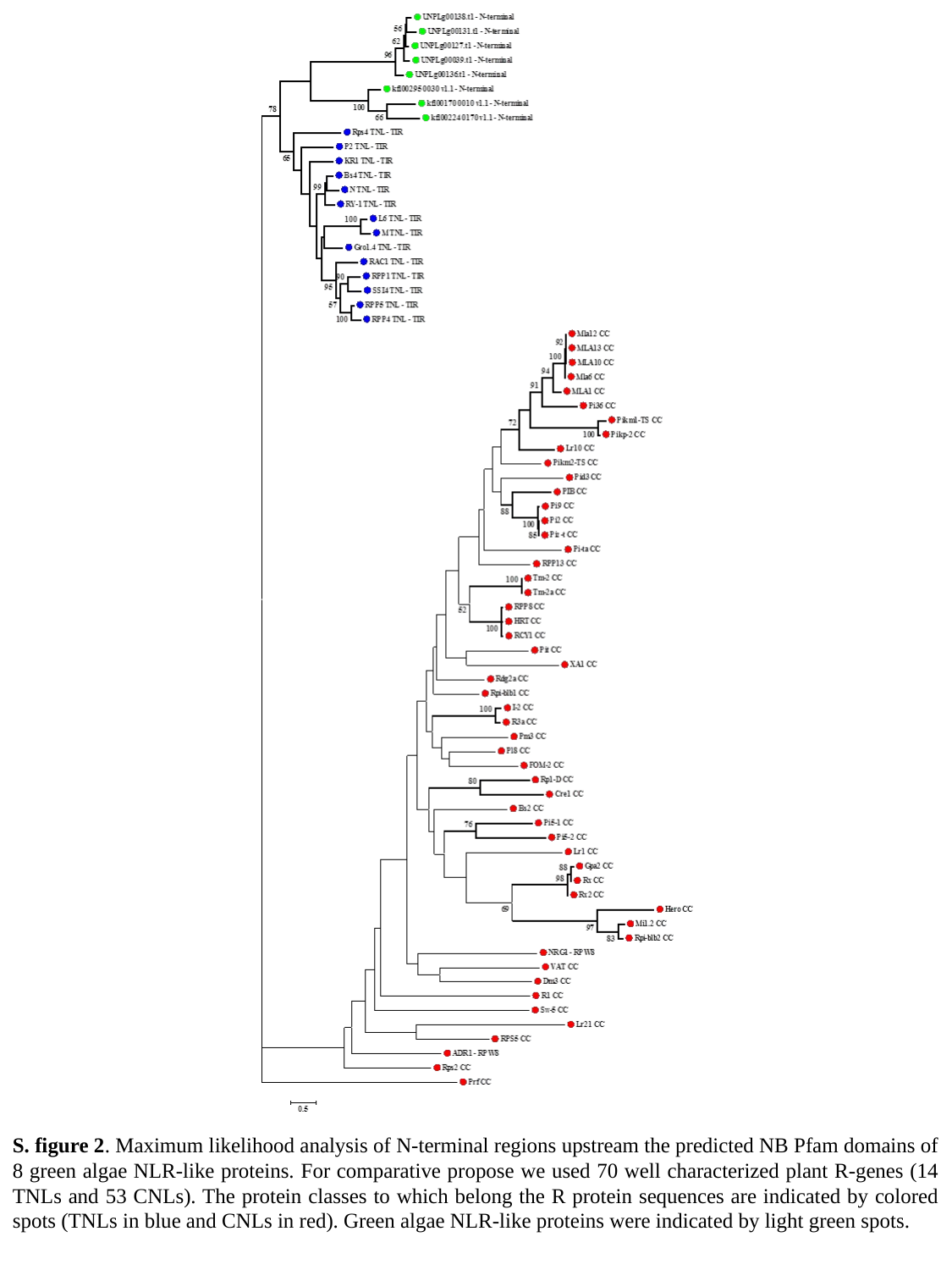

S. figure 2. Maximum likelihood analysis of N-terminal regions upstream the predicted NB Pfam domains of 8 green algae NLR-like proteins. For comparative propose we used 70 well characterized plant R-genes (14 TNLs and 53 CNLs). The protein classes to which belong the R protein sequences are indicated by colored spots (TNLs in blue and CNLs in red). Green algae NLR-like proteins were indicated by light green spots.

## Slide 3
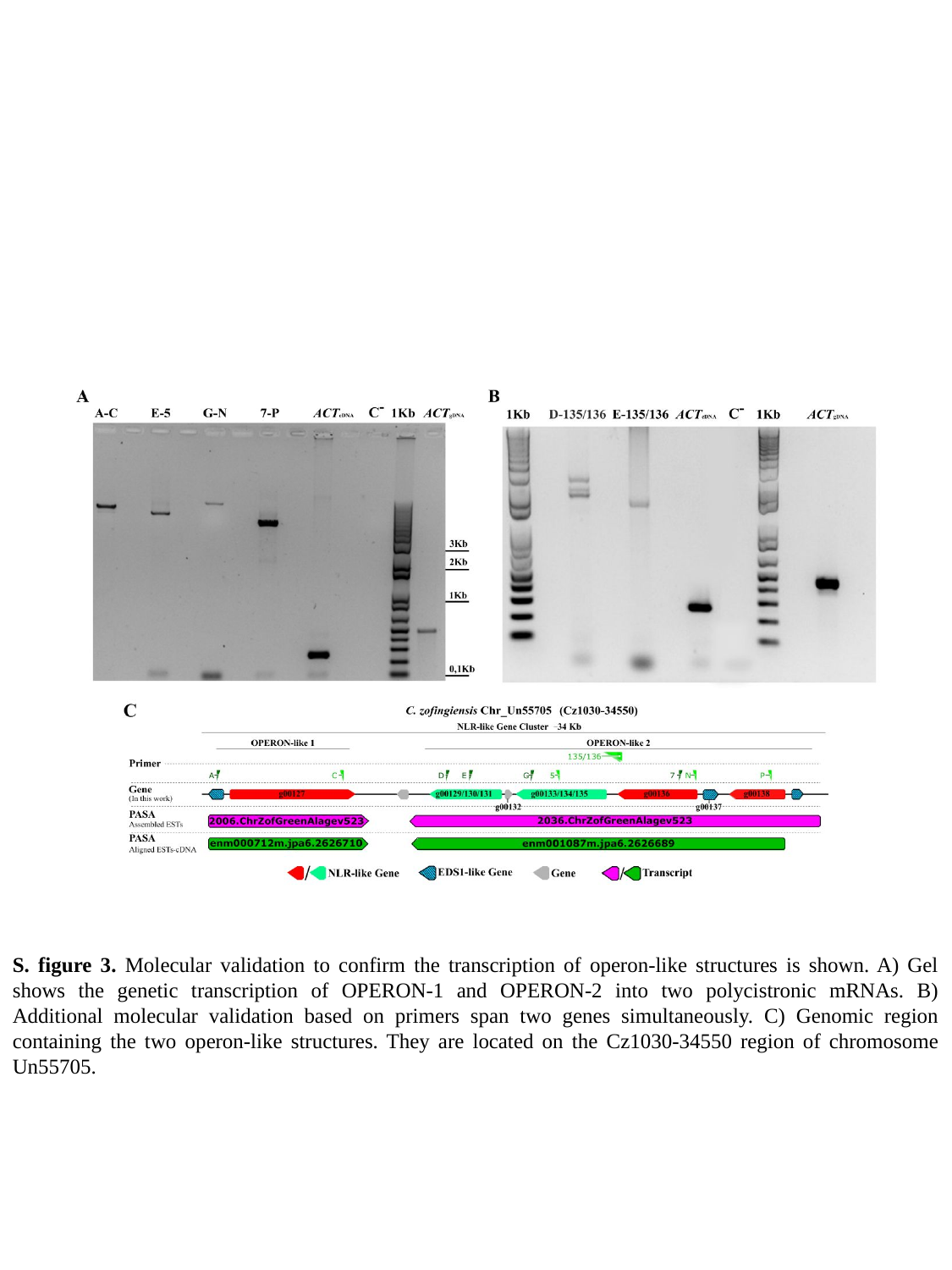

S. figure 3. Molecular validation to confirm the transcription of operon-like structures is shown. A) Gel shows the genetic transcription of OPERON-1 and OPERON-2 into two polycistronic mRNAs. B) Additional molecular validation based on primers span two genes simultaneously. C) Genomic region containing the two operon-like structures. They are located on the Cz1030-34550 region of chromosome Un55705.

## Slide 4
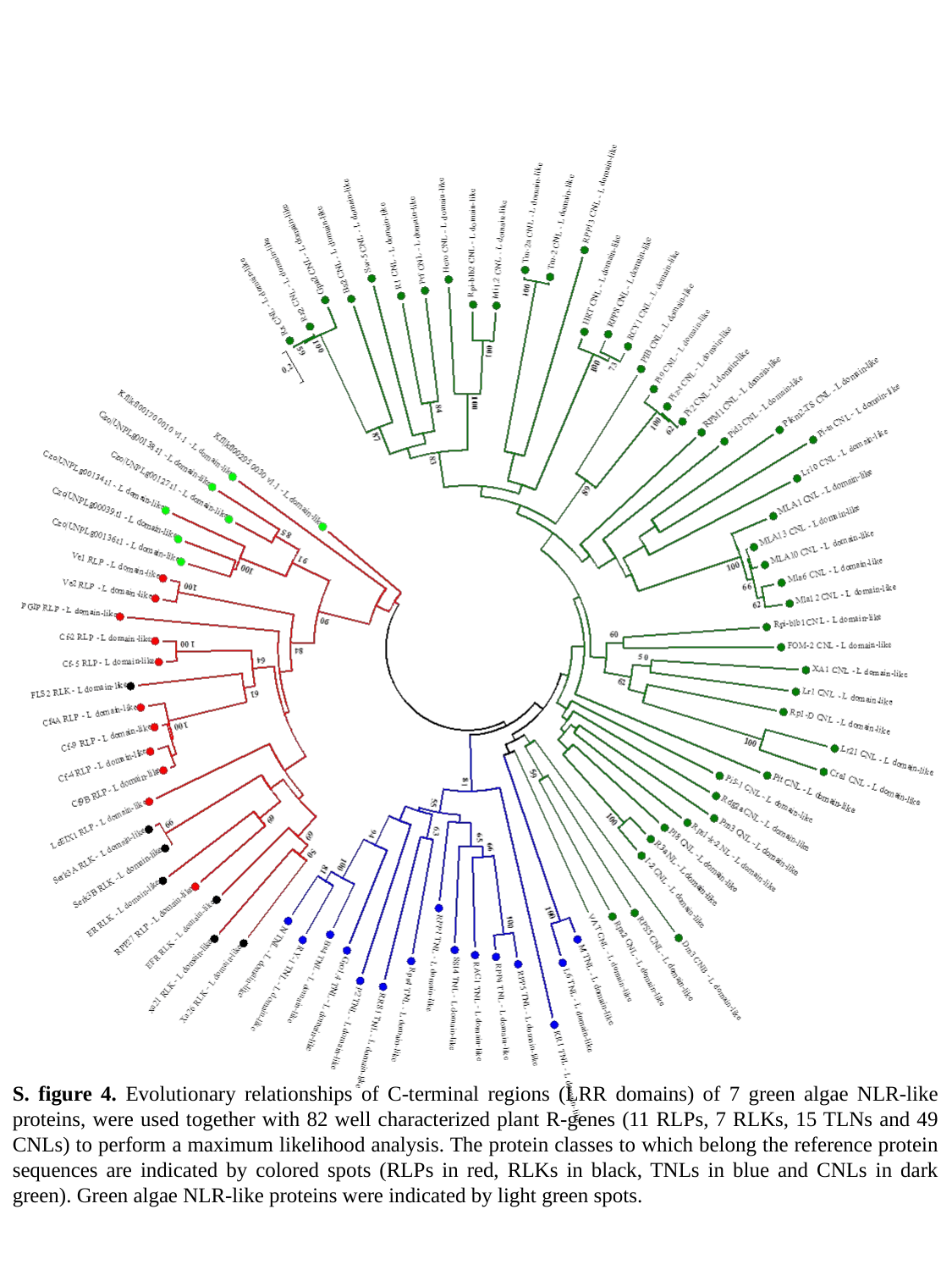

S. figure 4. Evolutionary relationships of C-terminal regions (LRR domains) of 7 green algae NLR-like proteins, were used together with 82 well characterized plant R-genes (11 RLPs, 7 RLKs, 15 TLNs and 49 CNLs) to perform a maximum likelihood analysis. The protein classes to which belong the reference protein sequences are indicated by colored spots (RLPs in red, RLKs in black, TNLs in blue and CNLs in dark green). Green algae NLR-like proteins were indicated by light green spots.
